# Changes and recovery of soil bacterial communities influenced by biological soil disinfestation as compared with chloropicrin-treatment

**DOI:** 10.1186/2191-0855-3-46

**Published:** 2013-08-17

**Authors:** Subrata Mowlick, Takashi Inoue, Toshiaki Takehara, Nobuo Kaku, Katsuji Ueki, Atsuko Ueki

**Affiliations:** 1 Faculty of Agriculture, Yamagata University, 1-23, Wakaba-machi, Tsuruoka 997-8555,Yamagata, Japan; 2 Yamaguchi Prefectural Technology Center for Agriculture and Forestry, 753-0214, Yamaguchi, Japan; 3 NARO Western Region Agricultural Research Center, 721-8514, Hiroshima, Japan

**Keywords:** Anaerobic bacteria, Biological soil disinfestation (BSD), Clone library, Chloropicrin, Clostridial group, Wilt disease of spinach

## Abstract

Soil bacterial composition, as influenced by biological soil disinfestation (BSD) associated with biomass incorporation was investigated to observe the effects of the treatment on the changes and recovery of the microbial community in a commercial greenhouse setting. Chloropicrin (CP) was also used for soil disinfestation to compare with the effects of BSD. The fusarium wilt disease incidence of spinach cultivated in the BSD- and CP-treated plots was reduced as compared with that in the untreated control plots, showing effectiveness of both methods to suppress the disease. The clone library analyses based on 16S rRNA gene sequences showed that members of the *Firmicutes* became dominant in the soil bacterial community after the BSD-treatment. Clone groups related to the species in the class *Clostridia*, such as *Clostridium saccharobutylicum*, *Clostridium tetanomorphum*, *Clostridium cylindrosporum*, *Oxobacter pfennigii*, etc., as well as *Bacillus niacini* in the class *Bacilli* were recognized as the most dominant members in the community. For the CP-treated soil, clones affiliated with the *Bacilli* related to acid-tolerant or thermophilic bacteria such as *Tuberibacillus calidus*, *Sporolactobacillus laevolacticus*, *Pullulanibacillus naganoensis*, *Alicyclobacillus pomorum*, etc. were detected as the major groups. The clone library analysis for the soil samples collected after spinach cultivation revealed that most of bacterial groups present in the original soil belonging to the phyla *Proteobacteria*, *Acidobacteria*, *Bacteroidetes*, *Gemmatimonadetes*, *Planctomycetes*, TM7, etc. were recovered in the BSD-treated soil. For the CP-treated soil, the recovery of the bacterial groups belonging to the above phyla was also noted, but some major clone groups recognized in the original soil did not recover fully.

## Introduction

Soil fumigation is an effective method to control soil-borne plant pathogens and soil is generally fumigated with chemicals before planting high-value cash crops. Although soil-borne pests were historically managed by methyl bromide (MeBr) fumigation, it has been banned due to its detrimental effects on the stratospheric ozone layer (Prather et al. 1984; Ristaino and Thomas 1997). MeBr is being phased out as it is still in limited use around the world. Chloropicrin (CP) as an alternative of MeBr has been widely used for soil fumigation in many countries of the world (Ibekwe et al. 2004; Takeuchi 2006). Since chemical fumigants are known to have broad biocidal activities in addition to pathogen control (Anderson 1993), many general soil microbes may also be affected according to their sensitivity to the chemicals. In contrast, biological soil disinfestation (BSD), mainly developed in the Netherlands (Blok et al. 2000) and Japan (Shinmura 2004) in organic agriculture, suppresses soil pathogens based on microbial activity on incorporated plant biomass under anaerobic conditions without using any chemical fumigants. BSD has attracted our interest as an environmentally friendly tool and a suitable alternative to chemical fumigation as well as other non-chemical methods for soil-borne disease management. The principles of BSD conducted in Japan include three steps: (1) incorporating plant biomass into the soil, (2) flooding the soil by irrigation, and (3) covering the soil surface with plastic films for about three weeks to induce reducing soil conditions (Shinmura 2000,  2004). The total process is completed within one month and crops can be planted after removing the plastic film and plowing the fields. Plant biomass sources such as *Brassica* spp., wheat bran, rice straw, rice bran, *Avena* spp., grasses, or other organic substances have been incorporated in BSD against soil-borne pests and diseases (Shinmura 2004; Goud et al. 2004; Momma 2008).

In our previous studies, using model experiments of BSD with wheat bran or *Brassica juncea* and *Avena strigosa* plants as biomass sources, we successfully controlled pathogen populations (*Fusarium oxysporum* f. sp. *lycopersici*, wilt pathogen of tomato and *F. oxysporum* f. sp. *spinacea*, wilt pathogen of spinach) incorporated into soil (Mowlick et al. 2012a, b). We analyzed the bacterial communities in the BSD-treated soil samples by polymerase chain reaction-denaturing gradient gel electrophoresis (PCR-DGGE) and clone library analysis based on the 16S rRNA gene sequences and it was shown that the community structures changed drastically in response to the treatments. Strictly anaerobic bacteria in the phylum *Firmicutes*, especially of the class *Clostridia*, became major bacterial groups in the soil communities together with some other aerobic or facultative anaerobic bacteria from the classes including *Bacilli* and *Gammaproteobacteria*. Considering large scale crop production, it is necessary to utilize results from field experiments to increase reliability of findings from model experiments. Thus, it is important for us to prove the efficacy of BSD for controlling soil-borne disease under field conditions (in practice) and to know the changes and recovery of the bacterial community structures in soil as affected by the treatments.

Soil microbial communities play various important roles in controlling soil fertility and plant yields (Pankhurst and Lynch 1994). Especially, the functioning of aerobic communities is essential for normal growth of most of the plants by maintaining nutrient availability and other synergistic activities. However, any fumigation treatment may cause disturbance of the soil microbial ecology such as elimination, proliferation or inclusion of specific microbial groups in the soil. Alterations in the microbial community composition may lead to changes that interfere with the functional diversity and, ultimately, the overall soil quality (De Boer et al. 2003). Therefore, the recovery of the original bacterial community structure after disinfestation treatments is likely important for proper soil functioning and crop cultivation in the treated soil. However, only a few studies have reported the impact of disinfestation methods, including CP-treatment, on soil bacterial communities and their recovery during cropping (Ibekwe et al. 2001; Hoshino and Matsumoto 2007).

The objectives of this study were to analyze the bacterial community structures in field soil after BSD-treatment in a greenhouse and to confirm the results obtained by the model experiments of our previous studies (Mowlick et al. 2012a, b). Furthermore, bacterial compositions were also analyzed for the treated soils after cultivation of spinach plants to investigate the recovery of the community during cropping. Similarly, CP-treated soil in the same greenhouse was also analyzed to compare with the results of the BSD-treatment. Clone library analysis (Maidak et al. 1999) based on 16S rRNA gene sequences was mainly carried out to determine the bacterial community compositions in these soil samples.

## Materials and methods

### Soil disinfestation and cultivation of spinach

A field experiment was conducted in a greenhouse in Abu-cho (34.3°N, 131.3°E), Yamaguchi, Japan, during June 2011. Soil was fine- textured yellow loamy soil (pH 5.5). Spinach had been continuously cultivated in the greenhouse for about ten years and natural infection of fusarium wilt disease (caused by *Fusarium oxysporum* f. sp*. spinaciae*) often occurred during the period. The number of treatments used in this experiment was three distributed in a randomized complete block design with three replications. The plot size for each treatment was 27.5 m^2^. For the control-treatment, no plant biomass or substances was incorporated into the soil. Wheat bran as BSD-treatment was incorporated at the rate of 1 kg/m^2^ using a rotary tiller. Irrigation water was applied to both treatments (100 kg/m^2^) to moisten the plots. The treated plots were covered with a double layer of plastic agricultural sheets with low gas permeability (Barrier Star film, TOKANKOUSAN Co. LTD; Sky Coat film, C.I. KASEI Co. LTD) (07 June). After three weeks of treatment (28 June), the sheets were removed and soil samples were immediately collected from the plots. Each soil sample (100 g) was obtained in triplicates from the upper 10 cm of soil depth and mixed well in sterile polyethylene bags. Similarly, an original field soil sample without any treatment was also collected. The soil samples collected were kept in a freezer (−20°C) immediately after the sampling and preserved there until use.

For the CP-treatment, CP was applied in the field soil using an injection machine (1DQ, AGRITECNO YAZAKI Co., LTD) attached at the rear of a 5 hp tractor (21 June). By the machine, 15-cm-deep holes (about 2 cm diameter) were made in every 900 cm^2^ (30 cm × 30 cm), and 3 ml of CP (80% solution) was injected into each hole, which was immediately filled with soil. Afterwards, the entire plot was covered with the transparent plastic sheets. The sheets were removed after a week and the plot was kept open to degas the fumigant until plowing. A soil sample was obtained on the same day (28 June) as described above.

All treated fields were plowed and spinach (cultivar *Mirage*) was seeded after a week (04 July). Pellet-type organic fertilizer containing fish meal (Kumiai Ube Yuki 100: N, 70 g/kg; P_2_O_5_, 40 g/kg; K_2_O, 10 g/kg) (MC FERTICOM, Co., LTD) was used as preplanting fertilizer to supply nitrogen of 20 g/m^2^ in each soil. The application amount was determined based on the nitrogen content measured by the microdiffusion method using samples extracted from each soil with 2 M KCl. Additional fertilizer was not applied throughout the cultivation. Spinach was sown using a seeding machine (Gonbe, Mukai Kogyo Inc.) with row-distance of 10 cm and intra-row space of 16 cm resulting in 63 stands/m^2^. Plants were watered as needed (about 10 minutes in the morning). Weeds were pulled by hand until two weeks after seeding and insect pests were mainly controlled using insect screens (4-mm mesh size) spread at all openings of the greenhouse. Insecticides were used when the urgent need arose. After one month of cultivation of spinach, soil samples were again taken from the plots (04 August).

### Measurements of disease incidence and soil analysis

Natural wilt disease incidence of spinach (caused by *F. oxysporum* f. sp. *spinacea*) was recorded by visual observation of total 240 plants (hill) per plot (from 12 different points for each plot) during the cropping year and fresh marketable (g/m^2^) yields were determined based on sampling of 40 plants from two different points in each plot. Temperature in soil (10 cm depth) covered with the sheets was monitored by data loggers during the disinfestation treatments. Air temperature inside the greenhouse was also recorded in the same way. Soil pH was measured in water (soil and distilled water at the ratio 1:2.5) by the glass electrode method immediately after the soil sampling. The concentration of volatile fatty acids (VFAs) in soil samples was measured by gas chromatography (Hitachi G-3900) as described previously (Mowlick et al. 2012a; Ueki et al. 1986) and the concentrations are shown in the text as those determined in the supernatant of slurry samples.

### PCR analysis of extracted DNA from soil

A composite sample (3 g) was prepared taking 1 g soil of each triplicate soil sample, from which 1 g soil was used for DNA extraction. DNA was extracted from all the soil samples using ‘Ultra Clean™ Soil DNA Isolation Kit’ (MO BIO Laboratories, Inc., Carlsbad, CA, USA) according to the manufacturer’s instructions. Bacterial 16S rRNA genes were PCR amplified using a primer set B27f (5’-AGA GTT TGA TYM TGG CTC AG-3’) and U1492r (5’-GGY TAC CTT GTT ACG ACT T -3’). The composition of PCR mixture (50 μl) and PCR amplification conditions were followed as described by Mowlick et al. (2012a). The PCR composition includes 1.25 U of *Taq* DNA polymerase, 15 mM Tris–HCl, 50 mM KCl, 1.5 mM MgCl_2_, 0.1% BSA, each dNTPs at a concentration of 200 μM, 0.25 μM of each primer, and 60–100 ng of template DNA. The PCR amplification conditions were: activation of the polymerase (95°C, 12 min) followed by 30 cycles consisting of denaturation (95°C, 1 min), annealing (50°C, 1 min), elongation (72°C, 1.5 min), and extension (72°C, 2 min). Amplified DNA fragments were confirmed after agarose gel electrophoresis and staining with ethidium bromide.

### Preparation of clone library and sequencing

Clone libraries were constructed to determine the bacterial community composition in the soil samples collected as described above. The names of clone libraries were designated as Pre-treatment, UTC (untreated control, or irrigated without wheat bran incorporation), UTC-Spinach (after spinach cultivation in UTC), BSD (wheat bran BSD-treatment), BSD-Spinach (after spinach cultivation in BSD-treated soil), CP, and CP-Spinach (after spinach cultivation in CP-treated soil).

The PCR products of DNA from these samples were purified using the QIAquick Gel Extraction Kit (Qiagen, Valencia, CA, and USA) and cloned into *Escherichia coli* JM109 competent cells following the instructions of pGEM-T Easy Vector Systems (Promega, Madison, WI, USA). The vector-harboring clones containing an insert of appropriate sizes (about 1500 bp) were cultured on Luria-Bertani (LB) plates by standard methods (Kaku et al. 2005). Sequence analysis (about 600 bp) was performed for a total of 96 clones from each soil sample using a sequence primer U515f (5’ GTG YCA GCM GCC GCG GTAA-3’) according to the Dye Terminator method using a capillary sequencer at Takara Co. Ltd.

### Database search, construction of trees and statistical analysis

Database searches for related 16S rRNA gene sequences were conducted with the BLAST program and GenBank database (Altschul et al. 1997). The ClustalW program of DDBJ was used to align the nucleotide sequences of the clone libraries. The phylogenetic trees were made using the neighbor-joining method (Saitou and Nei 1987) with the Njplot program in the ClustalW package (Thompson et al. 1994). Construction of OTUs (operational taxonomic unit at 97% similarity level), bootstrap resampling analysis, chimera checking, and rarefaction analysis were carried out as described previously (Mowlick et al. 2012b). The coverage of the clone libraries (*C*) was calculated from the equation *C* = 1-(*n*_1_/*N*); where *n*_1_ is the number of clones that occurred only once (frequency 1), and *N* is the total number of clones examined (Good 1953). Bacterial diversity was calculated using the Simpson’s index (*D*) by the function, *D* = 1-∑*n*(*n*-1)/(*N*(*N*-1)), where *n* = the total number of clones of a particular OTU and *N* = the total number of clones of all OTUs. Besides, Shannon-Wiener index (*H’*) was determined to compare the changes in diversity of bacterial communities within the libraries by the function: *H’* = −∑*P*_*i*_ log *P*_*i*,_ where the proportion of OTU i relative to the total number of OTU (*p*_*i*_) was calculated. All those indexes were calculated using online biodiversity calculator (http://www.alyoung.com/labs/biodiversity_calculator.html).

### Nucleotide sequence accession numbers

The nucleotide sequences obtained from the clone library analyses have been deposited in DDBJ/GenBank and assigned under the accession numbers AB734124-AB734385 (261 entries).

## Results

### Soil status and treatments effects on crop

The mean soil temperature (10 cm depth) during the soil treatments was 28.2°C with diurnal variations ranged from around 24.0°C (as the lowest in the nights) to 34.0°C (as the highest in the daytime). The mean air temperature in the greenhouse was 25.3°C with high diurnal variations (data not shown). Small amounts of VFAs, especially acetate (6.3 mmol/l) together with a trace amount of butyrate, were detected from the wheat bran-treated (BSD) soil sample collected at the end of the treatment. No VFAs were detected for other soil samples including those after cropping in the field. Soil pH values after treatments (UTC, BSD, and CP) were 6.2, 6.6, and 5.7, and those after cropping (UTC-Spinach, BSD-Spinach, and CP-Spinach) were 5.3, 5.6, and 5.9, respectively.

The untreated control plots showed a high incidence of spinach wilt (50.3%) but the BSD-treatment considerably reduced disease incidence (20.1% incidence) relative to the control (Table 1). However, the disease incidence was almost absent in the case of the CP-treated plot (0.5%). As for the fresh marketable yields, similar amounts were obtained from both treatment plots (887 and 767 g/m^2^ for BSD- and CP-treated, respectively), which were much higher than the control (436 g/m^2^).

**Table 1:** Wilt disease incidence and yields of spinach cultivated in the treated fields

**Soil treatment**	**Wilt disease**	**Yield of spinach**
	incidence (%)*	**(g/m**^**2**^)*
Untreated control	50.3 ± 17.2	436 ± 102
BSD	20.1 ± 15.7	887 ± 267
CP	0.5 ± 1.2	767 ± 281

* Mean ± SD.

### Changes in soil bacterial community composition by the disinfestation treatments

Clone library analysis was conducted for the soil samples collected. The affiliations of clone sequences from each soil sample are shown in Table 2 in relation to the percentages of abundance belonging to each phylum or class of the domain *Bacteria*.

**Table 2:** Composition profiles of phylogenetic groups of bacteria based on 16S rRNA gene sequences from the clone libraries

**Phylum or class**	**Clone library (% of abundance)**						
	**Pre-treatment**	**UTC**	**BSD**	**CP**	**UTC-spinach**	**BSD-spinach**	**CP-spinach**
*Alphaproteobacteria*	26.2	17.7	11.6	10.7	30.7	32.9	25.0
*Betaproteobacteria*	5.9	3.2	4.4	7.1	8.0	3.8	8.8
*Gammaproteobacteria*	11.9	14.5	8.7	-	14.7	8.9	7.4
*Deltaproteobacteria*	-	1.6	1.4	-	1.3	-	-
*Acidobacteria*	10.7	9.7	-	8.3	9.3	8.9	4.4
*Verrucomicrobia*	-	4.8	-	-	-	-	4.4
*Bacteroidetes*	13.1	8.1	-	-	1.3	10.1	2.9
*Planctomycetes*	3.6	4.8	2.9	1.2	4.0	6.3	-
*Firmicutes* (*Clostridia*)	-	1.6	24.6	-	-	2.5	-
*Firmicutes* (*Bacilli*)	3.6	3.2	34.8	53.6	1.3	3.8	4.4
*Gemmatimonadetes*	8.3	19.4	7.2	9.5	14.7	7.6	14.7
TM7	5.9	4.8	-	-	2.7	6.3	8.8
*Actinobacteria*	5.9	6.5	-	7.1	9.3	3.8	7.4
*Chloroflexi*	-	-	-	2.4	2.7	3.8	2.9
Others	4.8	-	4.4	-	-	1.3	8.8

-, not detected.

The pre-treatment soil showed diverse populations of different phylogenetic groups. The most abundant taxonomic group of the library was retrieved from the phylum *Proteobacteria* (44.0% of the total number of clones) distributed into three classes, *Alpha*-, *Beta*-, and *Gammaproteobacteria.* Other dominant phylogenetic groups were affiliated with the phyla *Bacteroidetes*, *Acidobacteria*, and *Gemmatimonadetes.*

The UTC (untreated control) showed diversified bacterial groups like those noted in the pre-treatment soil library but with decreased percentages of clones from the classes *Alpha*- and *Betaproteobacteria* as well as the phylum *Bacteroidetes*. The ratio of clones belonging to the phylum *Gemmatimonadetes* largely increased compared with the FS library along with members in the *Verrucomicrobia* phylum.

For both the BSD and CP (CP-treated) clone libraries, the phylogenetic compositions changed remarkably from that of the Pre-treatment soil library. The BSD clone library showed the presence of exclusively dominant bacterial groups in the phylum *Firmicutes* (59.4%, 34.8 and 24.6% from the *Bacilli* and the *Clostridia*, respectively). Members belonging to the *Acidobacteria, Bacteroidetes*, *Planctomycetes*, and TM7 phyla disappeared and those of the phyla *Proteobacteria* and *Gemmatimonadetes* decreased as compared with the Pre-treatment soil library. For the CP clone library clones belonging to the class *Bacilli* of the phylum *Firmicutes* accounted for more than half (53.6%) of the total clones. Also, a small number of clones were detected from the phyla *Proteobacteria*, *Gemmatimonadetes*, *Acidobacteria*, and *Actinobacteria*, whereas groups from other phyla were mostly absent from the library.

**Figure 1:**
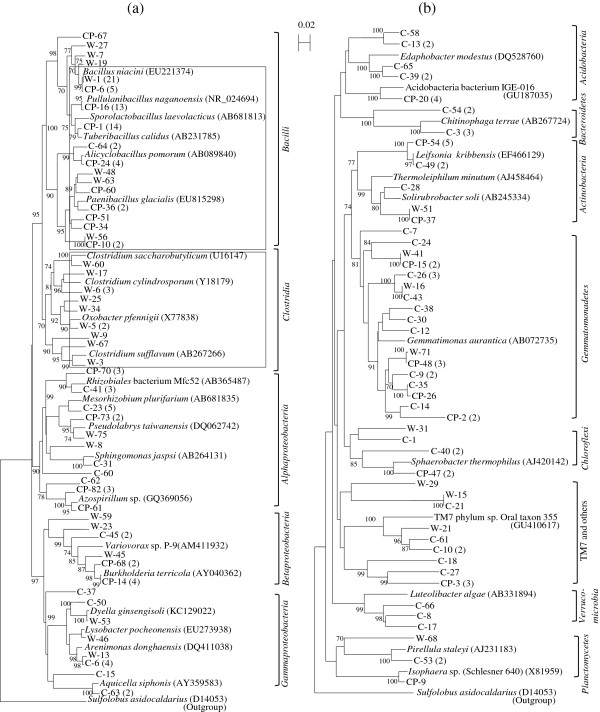
**Neighbor-joining tree showing the phylogenetic relationships of all OTUs derived from the three libraries (UTC, BSD and CP) of treated soil based on 16S rRNA gene sequences for (a) the phyla *Firmicutes *and *Proteobacteria*, and (b) other phyla (*Acidobacteria*, *Verrucomicrobia*, *Bacteroidetes*, *Planctomycetes*, *Gemmatimonadetes*, TM7 and others, *Actinobacteria*, and *Chloroflexi*).** Designation of clone names with C, W, and CP (deposited in the DDBJ/Genbank) corresponds to the clones belonging to the UTC, BSD and CP libraries, respectively. Bootstrap values (n = 1,000) above 70% are indicated at branch nodes. The scale bar represents 2% estimated difference in nucleotide sequence position. The name of each clone starts with designation of the respective clone libraries. As the outgroup, *Sulfolobus acidocaldarius* (D14053) (the domain *Archaea*) 16S rRNA gene sequence was used for both cases. Accession numbers of the species are shown in the parentheses. The phylogenetic groups of phyla and class levels are shown aside the close clusters. Numbers in the parentheses aside each clone name denote the number of clones assigned to the OTU. Each clone name without parenthesis represents one OTU with one clone. The squares marked with some clone clusters show the dominancy of major groups in the communities of the respective clone libraries.

The relationships of clones derived from the UTC, BSD, and CP libraries were examined in the phylogenetic tree that combined all clone groups (OTUs) from the three libraries (Figure 1). For the UTC library the clone groups related to *Mesorhizobium plurifarium* (the class *Alphaproteobacteria*), *Arenimonas donghaensis* (the class *Gammaproteobacteria*), and *Gemmatimonas aurantica* (the phylum *Gemmatimonadetes*) (90.8-97.0% of sequence similarity for 16S rRNA gene) were considered dominant members containing 4–5 clones. For the BSD library a clone group (OTU) related to *Bacillus niacini* (96.0-99.0%) in the *Bacilli*, aerobic or facultatively anaerobic spore-forming groups, appeared as the largest group (23 clones), and accounted for about 85% of the clones assigned to the *Bacilli* in the library. Clones belonged to the *Clostridia* class, strictly anaerobic spore-forming groups, were obtained only in the BSD library as shown in Table 1, and many species such as *Clostridium cylindrosporum* (92.2%), *Clostridium saccharobutylicum* (95.1%), and *Oxobacter pfennigii* (95.2%) were detected as the closest relatives of these clones.

For the CP library out of the clone groups affiliated with the *Bacilli,* a large number of clones were related to *Tuberibacillus calidus* or *Sporolactobacillus laevolacticus* (14 clones, 97.0-99.0%), *Pullulanibacillus naganoensis* (13 clones, 99.4%), *Alicyclobacillus pomorum* (4 clones, 98.0%), and *Paenibacillus* spp*.* (7 clones, 90.0-95.2%). Of these groups, the clones related to *Paenibacillus* spp*.* made a cluster with some clones from the BSD library, and a clone group closely related to *B. niacini* (5 clones, 99.0%) was also present in the BSD library. Although the high percentages of clones affiliated in the *Bacilli* were found in both the BSD and CP libraries, the members retrieved from that class in the two libraries were markedly different from each other. Also, a clone group closely related to *Burkholderia terricola* in the *Betaproteobacteria* (6 clones, 97.0-99.0%) was an almost unique dominant group (except for a clone in the BSD library) appearing in the CP library.

### Recovery of bacterial communities after cropping

The bacterial population compositions in soil after spinach cultivation in all treated plots were compared with the non-treated soil (Table 2). For the UTC-Spinach (after spinach cultivation in UTC) library the ratio of clones from the phylum *Proteobacteria* (especially for the *Alpha-* and *Betaproteobacteria*) increased as compared with those in the UTC library. Thus the UTC-Spinach library showed a similar composition profile as the Pre-treatment soil library except for the considerable decrease in the ratio of the members from the *Bacteroidete*s. For the BSD-Spinach (after spinach cultivation in BSD-treated soil) clone library members in the *Firmicutes* that dominated the BSD library declined markedly, while members of the *Proteobacteria* (45.6%) especially in the *Alphaproteobacteria* (32.9%) increased considerably as compared with those in the BSD library. Also, clones belonging to the *Acidobacteria*, *Bacteroidetes*, *Planctomycetes*, TM7, and *Actinobacteria* phyla reappeared in the BSD-Spinach library and the overall profile was almost the same as that of the Pre-treatment soil library except for the presence of clones from the *Chloroflexi* (3.8%) and the remaining minor members (2.5%) from the *Clostridia*.

For the CP-Spinach (after spinach cultivation in CP-treated soil) clone library clones of the *Bacilli* decreased enormously as compared with those in the CP library. Clones belonging to the phylum *Proteobacteria* (41.2%) especially in the class *Alphaproteobacteria* (25.0%) became dominant and the members of the class *Gammaproteobacteria* reappeared. Thus, it seems that a bacterial community with a similar profile to that of the Pre-treatment soil library returned in the CP-treated soil after spinach cultivation. However, clones from the *Planctomycetes* were absent and the ratios of the clones from both phyla *Acidobacteria* and *Bacteroidetes* were much lower as compared with those in the Pre-treatment soil library. Therefore, it appears that the community composition of the original soil did not fully recover in the spinach plot soil after the CP-treatment.

**Figure 2:**
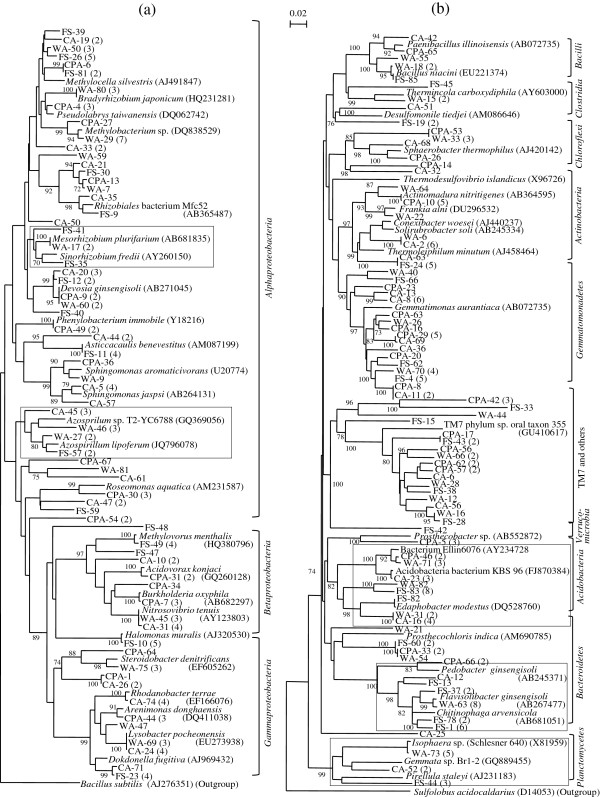
**Neighbor-joining trees showing the phylogenetic relationships of all OTUs derived from the clone libraries Pre-treatment, UTC-Spinach, BSD-Spinach, and CP-Spinach based on 16S rRNA gene sequences for (a) the phylum *Proteobacteria*; (b) other phyla (*Firmicutes*, *Acidobacteria*, *Verrucomicrobia*, *Bacteroidetes*, *Planctomycetes*, *Gemmatimonadetes*, TM7 and others, *Actinobacteria*, and *Chloroflexi*).** Designation of clone names with FS, CA, WA, and CPA (deposited in the DDBJ/Genbank) corresponds to the clones belonging to the Pre-treatment, UTC-Spinach, BSD-Spinach, and CP-Spinach clone libraries, respectively. The squares marked with some clone clusters show the recovery of the bacterial groups found in the original soil for the BSD-treatment but not for the CP. As the outgroups, 16S rRNA gene sequence of *Bacillus subtilis* DSM10 and *Sulfolobus acidocaldarius* (the domain *Archaea*) were used for **(a)** and **(b)**, respectively. Tree construction and other notifications are similar as described in Figure 1.

The recovery of bacterial communities after spinach cropping in the treated soil was examined by comparing the UTC-Spinach, BSD-Spinach, and CP-Spinach clone libraries with the Pre-treatment soil library in a combined phylogenetic tree (Figure 2). For most of the clone groups in the tree, clones from the four different libraries were placed in the same clusters indicating the recovery of the closely related bacterial species present in the original soil after spinach cultivation. However, some groups disappeared from the clusters consisting of the major members of the Pre-treatment soil library.

For the UTC-Spinach library, phylogenetic groups very similar to the Pre-treatment soil library were detected, although some members related to *Chitinophaga arvensicola* or *Flavisolibacter ginsengisoli* (the phylum *Bacteroidetes*) were not found in the library UTC-Spinach, as indicated with a low ratio of the clones in the phylum (Table 2). In the BSD-Spinach library, although two clones in the *Clostridia* were found, these were related to a thermophilic anaerobic bacterium, *Thermincola carboxydiphila*, and absent in the BSD library. Thus, typical clostridial clones found as the dominant groups in the BSD library had disappeared. The groups belonging to the phyla *Proteobacteria*, *Acidobacteria*, *Bacteroidetes*, *Planctomycetes*, *Gemmatimonadetes*, TM7, and *Actinobacteria* were present in almost all clusters consisting of clones from the Pre-treatment soil library except for a few clusters such as those related to *Halomonas muralis* (the class *Gammaproteobacteria*).

For the CP-Spinach library, the major clone groups from the *Bacilli* in the CP library were not found in the tree. Instead, most of the clones belonging to the phyla *Proteobacteria*, *Gemmatimonadetes*, TM7 and *Actinobacteria* made up the same clusters with the clones from the Pre-treatment soil library. However, clones related to *Azosprillum* sp. and *Mesorhizobium* sp. (the class *Alphaproteobacteria*), *H. muralis* (the class *Gammaproteobacteria*), *E. modestus* (the phylum *Acidobacteria*), *F. ginsengisoli* (the phylum *Bacteroidetes*), and *Pirellula staleyi* (the phylum *Planctomycetes*) as major groups in the Pre-treatment soil library, were only partially recovered or not recovered at all from the CP-Spinach soil sample.

### Bacterial diversity in soil

**Figure 3:**
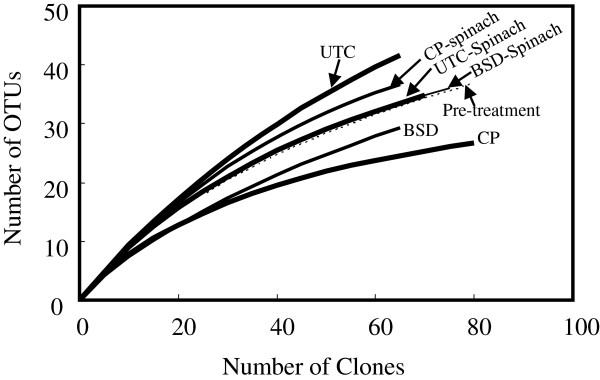
**Rarefaction curves for the 16S rRNA gene sequences from all clone libraries.** Libraries: Pre-treatment, UTC (untreated control), UTC-Spinach (after spinach cultivation in UTC), BSD (wheat bran BSD treatment), BSD-Spinach (after spinach cultivation in BSD-treated soil), CP, and CP-Spinach (after spinach cultivation in CP-treated soil).

The number of OTUs recognized for the clone libraries showed a varied proportion of abundance of clones and their diversity (Table 3). Rarefaction analysis based on the OTU clustering suggests that the curves for the Pre-treatment soil along with other control (UTC and UTC-Spinach) and spinach cultivated soil (BSD-Spinach, and CP-Spinach) were far from saturation. On the other hand, BSD and CP libraries seemed to near plateaus (Figure 3).

**Table 3:** Estimates of bacterial diversity in the soil samples obtained after the treatments and spinach cultivation

**Soil sample**	**Pre-treatment**	**UTC**	**BSD**	**CP**	**UTC-spinach**	**BSD-spinach**	**CP-spinach**
Total number of clones	85	66	68	80	75	79	67
Total number of OTUs	38	42	30	25	36	37	38
Coverage (%)	76.5	60.6	73.5	88.7	76.0	77.2	70.1
Simpson’s index	0.97	0.98	0.89	0.93	0.97	0.97	0.98
Shannon-Wiener index	4.87	5.19	4.01	4.10	4.87	4.87	5.05

The estimate of diversity in the communities suggests that the original field soil harbored the most diverse communities, and all soil treatments reduced the diversity. The community diversity recovered during cropping in the treated soil.

## Discussion

In the previous studies of our model experiments, BSD showed suppression of plant pathogens and anaerobic bacterial communities, while the *Clostridia* became dominant groups in the BSD-treated soil. In this study, we intended to confirm the BSD-effects under field conditions and compared them with a standard CP-treatment. It is always difficult to maintain desirable constant soil environments including temperature, moisture content, oxidation-reduction potential (ORP), etc., under field conditions. Controlled environment studies with smaller volumes in model experiments might enhance the production of VFAs or other products due to rapid and smooth growth of anaerobes as demonstrated in our previous studies (Mowlick et al. 2012b).

The increased soil temperature caused by covering the soil with plastic favored the rapid growth of microbes under a low O_2_ concentration and the development of anaerobiosis in the successful BSD treated soil. Although we did not measure the ORP values for the treated soil in this study, the detection of many members of the *Clostridia* class, which require strictly anaerobic conditions for growth, in the BSD library confirmed the development of anaerobic conditions in the soil. The accumulation of VFAs such as acetate and butyrate in soil has been described as an important aspect of BSD for the suppression of soil-borne pathogens (Momma et al. 2006; Mowlick et al. 2012a,  2012b), and VFAs were also detected from the wheat bran-treated BSD soil in this study. Although their concentrations were comparatively lower than those in soil of our previous model experiments, the result indicated that VFAs did accumulate in the BSD-treated field soil in practice. Fewer numbers of spinach plants were infected in the BSD-treated field as compared with the control plots. These results confirm our previous studies where fungal pathogens were suppressed by BSD treatments and thus demonstrate the effectiveness of BSD under field conditions. CP has been shown to reduce fungal pathogens effectively in many vegetable crops, including tomatoes and potatoes, and can thereby increase yield (Hutchinson 2005; Sydorovych et al. 2008). The present study suggests that the BSD-treatment could decrease the crop disease incidence in the field and increase the crop yield comparable to the CP-fumigation.

The clone library for the Pre-treatment soil contained diverse bacterial groups of different phyla and classes as identified by the analyses of non-treated soils in this and previous studies using soil from different areas in Japan. The bacterial composition in the control soil (library UTC) was similar to the profile of the Pre-treatment soil, and it was shown that the changes in the soil bacterial community caused by the control treatment (irrigation without plant biomass) were only minor. For the BSD (wheat bran)-treated soil, the increased ratios of the *Clostridia* and *Bacilli* groups in the library was consistent with the previous results of the model experiments and clearly indicated their dominance in the BSD-treated field soil. Bacterial species in the *Firmicutes* detected in this study usually produce spores, and thus they could survive in field soil under various conditions and proliferate in the BSD soil under the suitable growth conditions developed in the soil amended with abundant organic matter.

The detection of dominant clone groups related to the strictly anaerobic clostridial species, *C. saccharobutylicum*, *O. pfennigii*, *C. cylindrosporum*, *C. sufflavum*,etc., were also commonly found as related species in the BSD-treated soils in the model experiments, indicating their similar prevalence in the BSD-field soil. These species were believed to form various products including VFAs, alcohols or other compounds during decomposition of biomass (Krumholz and Bryant 1985; Rainey et al. 2009; Wiegel 2009), therefore suggesting the possible role of pathogenic suppression by the related species in BSD-treated soil in practice. A group closely related to *B. niacini* of the *Bacilli* class was the largest clone group in the BSD library. Since *Bacillus* species have been known to have broad functions, especially in various enzymatic activities (Wang et al. 2002; Hariprasad et al. 2011), the group is postulated to have a role in pathogen control.

Detailed analysis of bacterial communities in CP-treated soil has not been carried out except for some analyses by DGGE (Ibekwe et al. 2004; Hoshino and Matsumoto 2007). All these studies showed great changes in the bacterial communities in soil after the CP-treatment. The profile of the CP library obtained in this study suggested that some specific bacterial groups survived after the treatment that was not detected in the BSD-treated soil. Some species in the *Bacilli* have been recognized to survive chemical fumigation (Ibekwe et al. 2001) and related bacterial groups were detected as major and dominant groups in the CP library of this study. These major bacterial groups might have special physiological characteristics to survive in soil under the chemical treatment. Species closely related to the dominant clone groups such as *T. calidus* and *P. naganoensis*as thermophilic bacteria (Hatayama et al. 2006), *Sporolactobacillus* sp. and *P. illinoisensis* as acid-tolerant and spore-forming lactic acid producing bacteria (Hyronimus et al. 2000; Bayane et al. 2010), *A. pomorum* as thermo-acidophilic endospore-forming bacterium (Goto et al. 2003), and *B. oxyphila* as an acid tolerant bacterium (Otsuka et al. 2011) are rather uncommon as soil bacteria. These species are all acid-tolerant, acidophilic, or thermophilic bacteria as shown above, and thus the bacterial groups related to these species might have the specific ability to survive in the extreme environments created by the CP-treatment.

It may be possible that most of the bacterial cells present in the original field soil were killed by the CP-treatment together with pathogenic microbes, and the bacterial groups survived in the extreme condition were detected in the clone library CP. It seems invalid at present to conclude their prevalence in soil after the CP-treatment, since we did not quantify the number of bacteria in the soil samples.

The original field soil showed the most diversified population, while BSD- or CP-treatment reduced the diversity possibly due to the proliferation, elimination or dormancy of some restricted bacterial groups. However, bacterial communities had almost recovered to the original field condition after cultivation of spinach in the control and BSD-treated soils but not the CP-treated soil. As the BSD-treated soil was exposed to aerobic conditions by removing the covering sheets and plowing well in preparation for crop cultivation, the diverse aerobic bacteria might start to grow again. Also, the recovered bacterial groups might have special physiology to become re-activated in nature even after the treatment. For example, *P. taiwanensis*, designated as an aerobic bacterium closely related to the *Bradyrhizobium*, *Mesorhizobium*, and *Methylobacterium*species (the class *Alphaproteobacteria*) and closely related to major clone groups in the Pre-treatment soil library, might have universal distribution in soil and be able to survive the extreme treatment conditions (Kämpfer et al. 2006) and be able to recover after the treatment. Furthermore, the *Bacteroidetes* groups especially those related to the *Flavisolibacter* and *Chitinophaga* species are widely distributed in aerobic and anaerobic environments (Reichenbach and Dworkin 1991) and therefore could survive in BSD and recover again during crop cultivation.

Bacteria belonging to the phylum *Gemmatimonadetes* are frequently detected in environmental samples but *G. aurantiaca* was the only representative species isolated and characterized (DeBruyn et al. 2011). Candidate phylum TM7 is a phylogenetically independent phylum level lineage in the domain *Bacteria* that is widespread in the environment (Hugenholtz et al. 2001). These bacterial groups were detected in most of the clone libraries in this study and could easily recover in soil after spinach was cultivated in the treated soil. Anaerobic metabolic abilities are widely distributed among all major *Planctomycetes* lineages and carbohydrate fermentation or sulfur reduction should be possible mechanisms for growth and survival of the species in the group under the anaerobic treatment conditions (Elshahed et al. 2007). The *Planctomycetes* related bacterial groups recovered fully in the BSD-treated soil, whereas that was not the case in the CP-treated soil.

Stromberger et al. (2005) reported that CP and other chemical alternatives to MeBr have the potential to alter important microbial and enzymatic functions in soil. In this study, the clone library results clearly indicated that the soil bacterial community was greatly changed by the CP-treatment and most of the closest relatives of the clones were rather different from those of the BSD-treated library. It was reported that both ammonium-oxidizing and nitrate-oxidizing bacteria were severely affected by the CP-treatment (Tanaka et al. 2003). Although the original population seemed to have almost recovered during the crop cultivation in the CP-treated field, some major members in the phyla *Acidobacteria*, *Bacteroidetes*, and *Planctomycetes* were not detected in the CP-Spinach clone library. All these phylogenetic groups seem to be universal and thus important, but rather vulnerable constituents in the soil microbial ecosystem. The severe changes in soil bacterial communities by the CP-treatment may reduce the soil quality due to microbial imbalance. Therefore, the repeated application of CP as a soil fumigant may affect the sensitive microbial groups and destroy the original communities in soil permanently. Moreover, accelerated degradation is well known for single or repeated applications of chemical fumigant resulting in the rapid loss of the chemical’s efficacy to control soil-borne pathogens (Gamliel and Dotan 2009). Therefore CP as a soil fumigant may have potential risks in the world agriculture.

The results obtained in this study clearly demonstrated that all the proliferated bacterial groups by the BSD-treatment, especially the members of the *Firmicutes*, became minor groups under the detection limit of the clone library analysis and almost all the original bacterial communities in soil were recovered during cultivation of crop. BSD is a technology dependent on the microbial activities and the treatment itself does not aggressively kill microbial communities. BSD is a natural microbial phenomena reinforced artificially. Our results suggest that in terms of impacts on soil microbial communities, BSD may be a more sustainable option for soil disinfestation than CP treatment. However the results based on present clone library analysis may require some additional analytical methods such as DGGE, PLFA, or pyrosequencing analysis to examine treatments effects more strongly. This should be studied as a remaining subject for confirmation of the study.

## Competing interests

The authors declare that they have no competing interests.

## Authors’ contributions

SM carried out the molecular studies of soil bacterial communities and drafted the manuscript. TI carried out soil treatments, crop cultivation, and sampling of soil. TT participated in treatment design of BSD and carried out the technical guidance. NK conducted the technical guidance for cloning of the genes and drawing of the phylogenetic trees. KU participated in analysis of bacterial communities and contributed valuable suggestions to the study. AU supervised experimental design and revised the manuscript for submission. All authors read and approved the final manuscript.
